# Correction: Fundamentals and recent progress relating to the fabrication, functionalization and characterization of mesostructured materials using diverse synthetic methodologies

**DOI:** 10.1039/d0ra90055a

**Published:** 2020-05-11

**Authors:** Soroush Soltani, Nasrin Khanian, Umer Rashid, Thomas Shean Yaw Choong

**Affiliations:** Department of Chemical and Environmental Engineering, Universiti Putra Malaysia 43400 Selangor Malaysia soroush.soltaani@gmail.com csthomas@upm.edu.my +60 122635051; Department of Physics, Islamic Azad University Karaj Iran; Institute of Advanced Technology, Universiti Putra Malaysia 43400 Selangor Malaysia

## Abstract

Correction for ‘Fundamentals and recent progress relating to the fabrication, functionalization and characterization of mesostructured materials using diverse synthetic methodologies’ by Soroush Soltani *et al.*, *RSC Adv.*, 2020, **10**, 16431–16456, DOI: 10.1039/D0RA00440E.

The authors regret that there was an error in the author list in the original article. Thomas Shean Yaw Choong should have been listed as a corresponding author. The amended author list and contact details are shown above.

The Royal Society of Chemistry apologises for these errors and any consequent inconvenience to authors and readers.

## Supplementary Material

